# Opposing Roles of DCs and iNKT Cells in the Induction of Foxp3 Expression by MLN CD25^+^CD4^+^ T Cells during IFNγ-Driven Colitis

**DOI:** 10.3390/ijms232315316

**Published:** 2022-12-05

**Authors:** Sung Won Lee, Hyun Jung Park, Luc Van Kaer, Seokmann Hong

**Affiliations:** 1 Department of Integrative Bioscience and Biotechnology, Institute of Anticancer Medicine Development, Sejong University, Seoul 05006, Republic of Korea; 2 Department of Pathology, Microbiology and Immunology, Vanderbilt University School of Medicine, Nashville, TN 37232, USA

**Keywords:** iNKT cells, IFNγ, Treg cells, dendritic cells, CD25^+^CD4^+^ T cells

## Abstract

We have previously shown that a deficiency of CD1d-restricted invariant natural killer T (iNKT) cells exacerbates dextran sulfate sodium (DSS)-induced colitis in Yeti mice that exhibit IFNγ-mediated hyper-inflammation. Although iNKT cell-deficiency resulted in reduced Foxp3 expression by mesenteric lymph node (MLN) CD4^+^ T cells in DSS-treated Yeti mice, the cellular mechanisms that regulate Foxp3 expression by CD25^+^CD4^+^ T cells during intestinal inflammation remain unclear. We found that Foxp3^−^CD25^+^CD4^+^ T cells expressing Th1 and Th17 phenotypic hallmarks preferentially expanded in the MLNs of DSS-treated Yeti/CD1d knockout (KO) mice. Moreover, adoptive transfer of Yeti iNKT cells into iNKT cell-deficient Jα18 KO mice effectively suppressed the expansion of MLN Foxp3^−^CD25^+^CD4^+^ T cells during DSS-induced colitis. Interestingly, MLN dendritic cells (DCs) purified from DSS-treated Yeti/CD1d KO mice promoted the differentiation of naive CD4^+^ T cells into Foxp3^−^CD25^+^CD4^+^ T cells rather than regulatory T (Treg) cells, indicating that MLN DCs might mediate Foxp3^+^CD25^+^CD4^+^ T cell expansion in iNKT cell-sufficient Yeti mice. Furthermore, we showed that Foxp3^−^CD25^+^CD4^+^ T cells were pathogenic in DSS-treated Yeti/CD1d KO mice. Our result suggests that pro-inflammatory DCs and CD1d-restricted iNKT cells play opposing roles in Foxp3 expression by MLN CD25^+^CD4^+^ T cells during IFNγ-mediated intestinal inflammation, with potential therapeutic implications.

## 1. Introduction

Interferon-gamma (IFNγ), a type II interferon, is produced by natural killer (NK) cells, natural killer T (NKT) cells, CD8^+^ T cells, and CD4^+^ T-helper 1 (Th1) cells [1]. An evolutionarily conserved AU-rich element (ARE) region in the 3′-untranslated region (UTR) of the IFNγ mRNA regulates its stability, influencing protein synthesis in cytotoxic effector cells, such as NK and CD8^+^ T cells [2,3]. Increased expression of IFNγ highly correlates with the severity of chronic inflammatory disorders, including systemic lupus erythematosus and inflammatory bowel disease (IBD) [1]. Moreover, mice in which the ARE region of the IFNγ gene is deleted exhibit increased IFNγ mRNA stability and develop typical autoimmune symptoms characteristic of Th1-type hyperimmune responses [4]. Polymorphisms in the IFNγ gene are associated with disease severity in major forms of human inflammatory bowel diseases (IBDs), such as Crohn’s disease and ulcerative colitis [5].

Mice carrying one allele of a yellow fluorescent protein (YFP) cytokine reporter knocked into the 3′UTR of their IFNγ gene (heterozygous Yeti mice) exhibit autoinflammatory symptoms (i.e., splenomegaly and myeloproliferation), which have been attributed to chronically elevated IFNγ transcript levels [6]. Homozygous Yeti mice show organ failure and early mortality between 6 and 8 weeks of age [6]. Abnormal IFNγ secretion by Yeti CD4^+^ T cells is caused by overstimulation of glycolytic enzyme genes (i.e., lactate dehydrogenase A) [7]. In heterozygous Yeti mice, high levels of IFNγ can increase the number of cytotoxic CD8^+^ T cells expressing NK cell receptors (i.e., NK1.1 and NKG2D), resulting in increased susceptibility to dextran sulfate sodium (DSS)-induced colitis [8,9].

Invariant NKT (iNKT) cells recognize lipids presented by the MHC class I–like molecule CD1d and rapidly secrete immunosuppressive cytokines, such as TGFβ and IL10, in addition to inflammatory cytokines, such as IFNγ and TNFα [10,11,12,13,14]. Thus, iNKT cells can play both protective and pathogenic roles in autoimmune and allergic disorders [8,9,15,16,17,18,19]. For example, crosstalk between iNKT cells and type 3 innate lymphoid cells (ILC3s) in the mesenteric lymph nodes (MLNs) controls IFNγ-mediated intestinal inflammation [9]. In addition, it is well established that IL2 derived from activated iNKT cells promotes expansion of regulatory T (Treg) cells [8,15,20] and proliferation of memory Th1 and Th2 cells [21]. In particular, the iNKT-Treg cell axis plays a regulatory role in maintaining homeostasis in adipose tissue, skin, and the intestine [8,15,20].

Although many immune cells (i.e., iNKT cells, Treg cells, and ILC3 cells) have been implicated in controlling IFNγ-mediated intestinal inflammation in Yeti mice [8,9], the role of dendritic cells (DCs) in IFNγ-dysregulated intestinal inflammation has yet to be fully elucidated. Therefore, in this study, we investigated the immunoregulatory effects of DCs and iNKT cells on CD4^+^ T cell polarization in the MLNs during DSS-induced intestinal inflammation.

## 2. Results

### 2.1. Dysregulated IFNγ Expression in the Absence of iNKT Cells Causes Alterations in Foxp3 Expression by CD25^+^CD4^+^ T Cells in MLNs but Not Spleen during DSS-Induced Colitis

**Figure 1:**
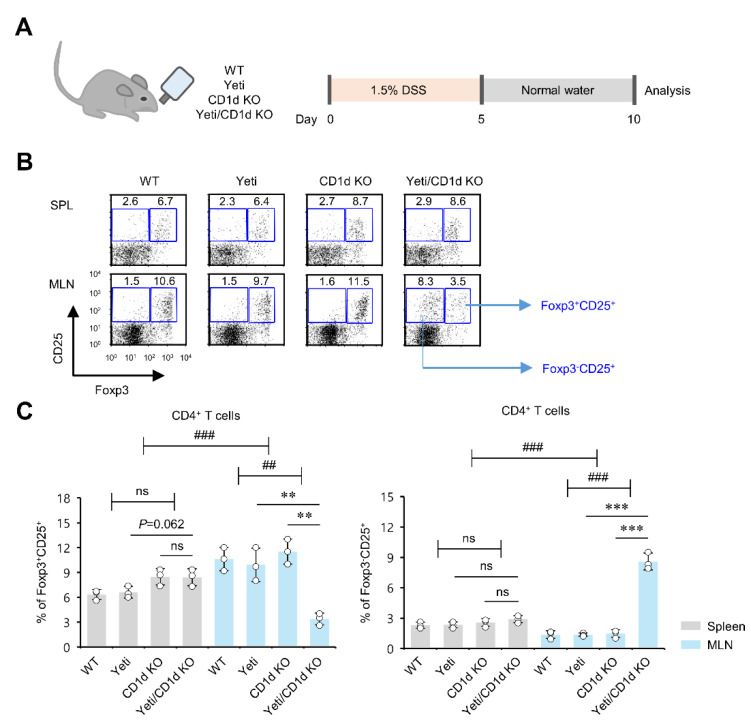
Dysregulated IFNγ expression in the absence of iNKT cells causes alterations in Foxp3 expression by CD25^+^CD4^+^ T cells in the MLNs, but not the spleen during DSS-induced colitis. (**A**) Experimental outline. WT, Yeti, CD1d KO, and Yeti/CD1d KO mice were treated with 1.5% DSS as described in Materials and Methods. (**B**) On day 10, the spleen and MLNs were obtained from these mice. The percentages of Foxp3^+^CD25^+^ and Foxp3^−^CD25^+^ subpopulations gated on CD4^+^ T cells from the spleen and MLNs of each group were measured by flow cytometry. Representative data (**B**) and their summary (**C**) are shown. The mean values ± SD (*n* = 3; per group in the experiment; Student’s *t*-test; ** *p* < 0.01, *** *p* < 0.001) are shown. Two-way ANOVA (Yeti × iNKT) showed an interaction between these two factors (^##^ *p* < 0.01, ^###^ *p* < 0.001). Two-way ANOVA (genotype × tissue) showed an interaction between these two factors (^###^ *p* < 0.001). One representative experiment from two experiments is shown. ns, not significant.

Yeti mice on the C57BL/6 (B6) genetic background (hereafter Yeti mice) display autoinflammatory syndromes due to abnormal IFNγ expression [6,7,8,9,22]. We have recently reported that iNKT cells play a central role in alleviating IFNγ-mediated intestinal inflammation in these animals [8,9,23]. Employing wild-type (WT), CD1d knockout (KO), Yeti, and Yeti/CD1d KO mice, we first confirmed the protective role of iNKT cells in DSS-induced and IFNγ-mediated intestinal inflammation (Figure S1). Since it has been reported that colonic Foxp3^+^ Treg cells play a critical role in suppressing the excessive infiltration of inflammatory immune cells into the colon during colitis [24], we next examined whether severe DSS-induced colitis observed in iNKT cell-deficient Yeti/CD1d KO mice may be attributed to altered profiles of Treg populations. To address this issue, we compared the Treg cell population in the spleen and MLNs among WT, CD1d KO, Yeti, and Yeti/CD1d KO mice after DSS treatment (Figure 1A). Intriguingly, DSS-treated Yeti/CD1d KO mice contained fewer Foxp3^+^CD25^+^ Treg cells in the MLNs but not spleen when compared with WT, Yeti, and CD1d KO mice. In sharp contrast, the frequency of Foxp3^−^CD25^+^CD4^+^ T cells (which likely include pathogenic T cells) was markedly increased in Yeti/CD1d KO mice (Figure 1B,C). Furthermore, we found that the frequency of Foxp3^−^CD25^+^CD4^+^ T cells was slightly but significantly increased in the MLN but not the spleen of untreated Yeti/CD1d KO mice, indicating that these mice may undergo low-grade inflammation in the MLN under steady-state conditions (Figure S2).

### 2.2. The MLN Foxp3^−^CD25^+^CD4^+^ T Cells from DSS-Treated Yeti/CD1d KO Mice Exhibit a Pathogenic Effector Phenotype

**Figure 2:**
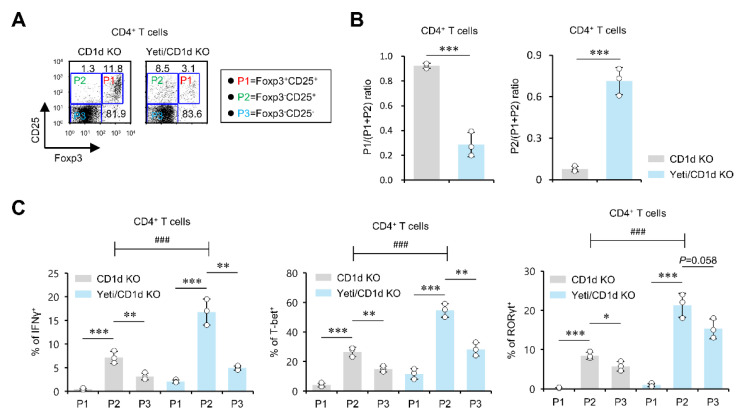
The MLN Foxp3^−^CD25^+^CD4^+^ T cells from DSS-treated Yeti/CD1d KO mice exhibit a pathogenic effector phenotype. (**A**–**C**) MLNs were obtained from CD1d KO and Yeti/CD1d KO mice on day ten after 1.5% DSS treatment. (**A**) The percentages of Foxp3^+^CD25^+^ (P1), Foxp3^−^CD25^+^ (P2), and Foxp3^−^CD25^−^ (P3) populations among MLN CD4^+^ T cells were evaluated by flow cytometry. (**B**) The ratio of the P1 or P2 to the CD25^+^CD4^+^ (P1 + P2) population was evaluated by flow cytometry. (**C**) Intracellular expression of IFNγ, T-bet, and RORγt on P1, P2, and P3 populations of MLN CD4^+^ T cells from CD1d KO and Yeti/CD1d KO mice was evaluated by flow cytometry. The mean values ± SD (*n* = 3; per group in the experiment; Student’s *t*-test; * *p* < 0.05, ** *p* < 0.01, *** *p* < 0.001) are shown. Two-way ANOVA (Yeti × iNKT) showed an interaction between these two factors (^###^ *p* < 0.001). One representative experiment from two experiments is shown.

It has been previously reported that the Foxp3^−^CD25^+^ subset among CD4^+^ T cells displays an activated phenotype with IFNγ and IL17 expression [25]. To investigate whether MLN CD4 T cells in Yeti/CD1d KO mice display proinflammatory phenotypes, MLN CD4^+^ T cells were subdivided into three populations depending on their expression status of Foxp3 and CD25 molecules as follows: Foxp3^+^CD25^+^ (P1), Foxp3^−^CD25^+^ (P2), or Foxp3^−^CD25^−^ (P3) CD4^+^ T cell subpopulations. Yeti/CD1d KO mice showed fewer Foxp3^+^CD25^+^ (P1) cells but increased Foxp3^−^CD25^+^ (P2) cells in the MLNs compared to control CD1d KO mice (Figure 2A). Interestingly, we found that Yeti/CD1d KO mice show remarkably opposite patterns of Foxp3 expression between P1 and P2 populations compared to the control mice, which implies that changes in Foxp3 expression occurred in both the P1 and P2 populations (Figure 2B). Next, we compared the levels of IFNγ, Th1 transcription factor T-bet, and Th17 transcription factor RORγt expression in these three CD4^+^ T cell subpopulations (P1, P2, or P3) from Yeti/CD1d KO mice to identify their functional properties. Whereas the Foxp3^+^CD25^+^ (P1) population showed significantly lower expression of IFNγ, T-bet, and RORγt, the Foxp3^−^CD25^+^ (P2) population displayed substantially higher expression of these molecules compared to both P1 and P3 populations (Figure 2C). Moreover, we found that Yeti/CD1d KO mice exhibited significantly increased expression of IFNγ, T-bet, and RORγt in the MLN P2 population compared with CD1d KO mice (Figure 2C). Our results demonstrate that increases in the prevalence of Foxp3^−^CD25^+^CD4^+^ pathogenic effector T cells correlate with increased susceptibility to DSS-induced colitis in Yeti/CD1d KO mice.

### 2.3. MLN iNKT Cells Play a Critical Role in Regulating Foxp3 Expression by CD25^+^CD4^+^ T Cells during DSS-Induced Colitis

**Figure 3:**
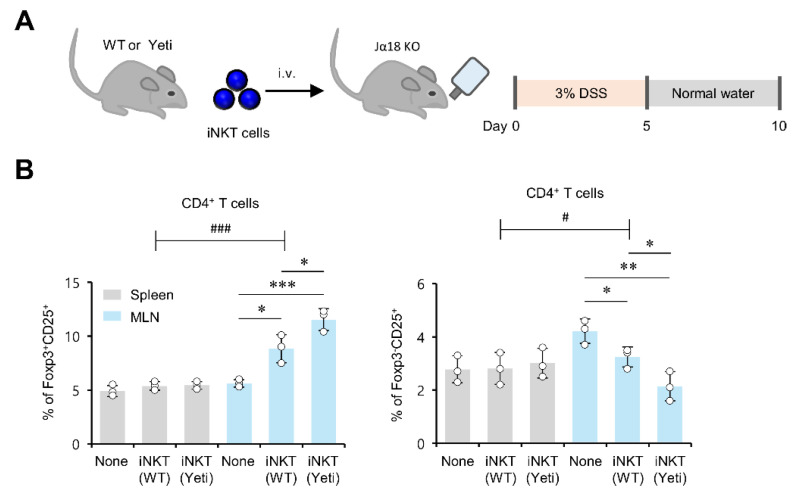
MLN iNKT cells play a critical role in regulating Foxp3 expression by CD25^+^CD4^+^ T cells during DSS-induced colitis. (**A**) iNKT cell-deficient Jα18 KO mice were adoptively transferred with either WT or Yeti MLN iNKT cells (2 × 10^5^) and subsequently treated with 3% DSS as described in Materials and Methods. (**B**) The frequencies of Foxp3^+^CD25^+^ and Foxp3^−^CD25^+^ subsets among CD4^+^ T cells in the spleen and MLNs from these mice were determined by flow cytometry on day 10. The mean values ± SD (*n* = 3; per group in the experiment; Student’s *t*-test; * *p* < 0.05, ** *p* < 0.01, *** *p* < 0.001) are shown. Two-way ANOVA (Yeti × iNKT) showed an interaction between these two factors (^#^ *p* < 0.05, ^###^ *p* < 0.001). One representative experiment from two experiments is shown.

Interactions between iNKT cells and Treg cells have been implicated in controlling autoimmune diseases [26]. Our previous study demonstrated that iNKT cells derived from Yeti mice are superior to iNKT cells derived from WT mice in inducing the activation of IL22-producing ILC3s during DSS-mediated colitis, and such an effect of Yeti iNKT cells closely correlated with their capacity to produce increased amounts of IL2, IL4, and IL9 [9]. Thus, to examine the effects of MLN iNKT cells on the generation of either protective Foxp3^+^CD25^+^CD4^+^ Treg cells or pathogenic Foxp3^−^CD25^+^CD4^+^ T cells, we explored many potential alterations of CD4^+^ T cells in DSS-treated Jα18 KO (iNKT cell-deficient) mice that received an adoptive transfer of MLN iNKT cells purified from either WT or Yeti mice (Figure 3A). Interestingly, the transfer of Yeti MLN iNKT cells resulted in a reduction in colitis severity in Jα18 KO mice compared to WT iNKT cells (Figure S3). MLN-derived WT iNKT cells efficiently upregulated the frequency of Foxp3^+^CD25^+^CD4^+^ Treg cells but downregulated the frequency of Foxp3^−^CD25^+^CD4^+^ T cells compared to the nontransferred group. Furthermore, MLN-derived Yeti iNKT cells significantly enhanced the Foxp3^+^CD25^+^CD4^+^ Treg cell population but, remarkably, also suppressed pathogenic Foxp3^−^CD25^+^CD4^+^ T cells even more than MLN-derived WT iNKT cells (Figure 3B). Collectively, these data indicate that iNKT cells play a critical role in regulating Foxp3 expression by CD25^+^CD4^+^ T cells and thereby determine the fate of these CD25^+^CD4^+^ T cells into either protective or pathogenic ones during DSS-induced intestinal inflammation.

### 2.4. Dysregulated IFNγ Production in the Absence of iNKT Cells Induces MLN DCs to Produce Pro-Inflammatory Cytokines during DSS-Induced Colitis

**Figure 4:**
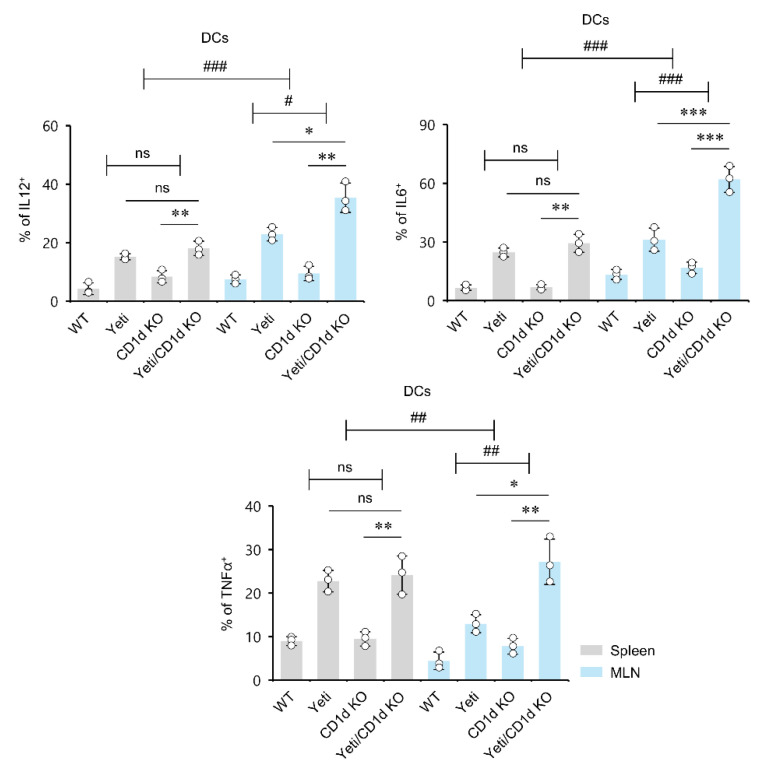
MLN Dysregulated IFNγ expression in the absence of iNKT cells causes DCs to produce proinflammatory cytokines in the MLNs during DSS-induced colitis. The spleen and MLNs were obtained from the indicated mice, as shown in Figure 1. Cytokine production (IL12, IL6, and TNFα) by splenic and MLN CD11c^+^ DCs was determined by flow cytometry on day 10. The mean values ± SD (*n* = 3; per group in the experiment; Student’s *t*-test; * *p* < 0.05, ** *p* < 0.01, *** *p* < 0.001) are shown. Two-way ANOVA (Yeti × iNKT) showed an interaction between these two factors (^#^ *p* < 0.05, ^##^ *p* < 0.01, ^###^ *p* < 0.001). Two-way ANOVA (genotype × tissue) showed an interaction between these two factors (^##^ *p* < 0.01, ^###^ *p* < 0.001). One representative experiment from three experiments is shown. ns, not significant.

During conditions of intestinal inflammation, DCs in the MLNs failed to induce Foxp3^+^ Treg cells but instead promoted the differentiation of Th1 cells, consequently contributing to IBD pathology [27]. Therefore, to examine whether MLN DCs from Yeti/CD1d KO mice show proinflammatory phenotypes during DSS-induced colitis, we compared proinflammatory cytokine production (i.e., IL12, TNFα, and IL6) by both splenic DCs and MLN DCs from WT, Yeti, CD1d KO, and Yeti/CD1d KO mice during DSS treatment. Compared to the non-Yeti groups, Yeti-expressing mice showed an increased frequency of DCs producing proinflammatory cytokines (i.e., IL12, TNFα, and IL6) in both the spleen and MLNs at day 10 after DSS treatment. In addition, iNKT cell-deficient Yeti (Yeti/CD1d KO) mice displayed much higher production of these cytokines in the MLN DCs compared with Yeti mice, suggesting that MLN iNKT cells play a crucial role in controlling proinflammatory DCs (Figure 4).

### 2.5. Pro-Inflammatory DCs from Yeti/CD1d KO Mice Induce the Differentiation of Foxp3^−^CD25^+^CD4^+^ Effector Cells and Antagonize Treg Cell Differentiation

**Figure 5:**
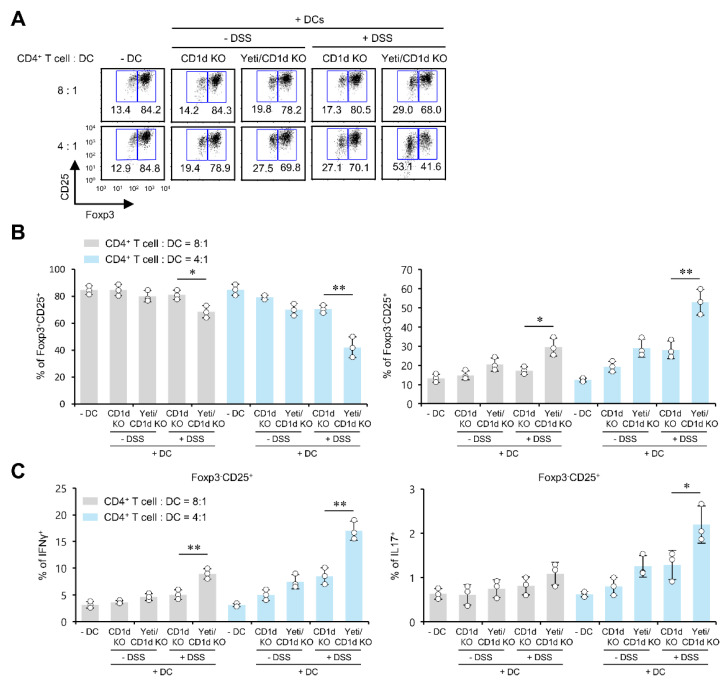
Proinflammatory DCs from Yeti/CD1d KO mice induce the differentiation of Foxp3^−^CD25^+^CD4^+^ effector cells and antagonize Treg cell differentiation. (**A**–**C**) Naive CD4^+^CD62L^+^ T cells were isolated from Jα18 KO mice. These naive T cells (1 × 10^5^ cells/well) were cocultured under Treg-polarizing conditions with MLN DCs (2.5 × 10^4^ or 1.25 × 10^4^ cells/well) purified from either CD1d KO or Yeti/CD1d KO mice treated or untreated with 1.5% DSS. (**A**,**B**) The frequencies of Foxp3^+^CD25^+^ and Foxp3^−^CD25^+^ subsets among CD4^+^ T cells were analyzed on day 5. (**C**) The intracellular expression of IFNγ and IL17 in Foxp3^−^CD25^+^CD4^+^ T cells of each group was analyzed by flow cytometry on day 5. The mean values ± SD (*n* = 3; per group in the experiment; Student’s *t*-test; * *p* < 0.05, ** *p* < 0.01) are shown. One representative experiment from two experiments is shown.

Proinflammatory DCs can confer inhibitory effects against the differentiation of Treg cells [28]. Therefore, to investigate whether the downregulation of Treg cells in Yeti/CD1d KO mice during DSS-induced colitis might be caused by altered DC functions, we evaluated the effects of DCs on Treg cell differentiation in these mice. For this purpose, we employed an in vitro inducible Treg (iTreg) cell differentiation assay. First, we measured the extent of iTreg cell differentiation after coculture of naive CD4^+^ T cells with total MLN DCs derived from either CD1d KO or Yeti/CD1d KO mice treated with or without DSS. We found an increased Foxp3^−^CD25^+^ population but a decreased Foxp3^+^CD25^+^ (iTreg) population in cocultures of CD4^+^ T cells with DCs from DSS-treated Yeti/CD1d KO mice compared with DSS-treated CD1d KO mice (Figure 5A,B). As the Foxp3^−^CD25^+^ population was significantly increased after coculture with DCs from Yeti/CD1d KO mice, we next examined cytokine production (i.e., IFNγ and IL17) by the Foxp3^−^CD25^+^ population. We found that the production of IFNγ and IL17 by Foxp3^−^CD25^+^ cells was significantly increased in the presence of Yeti/CD1d KO DCs compared with CD1d KO DCs (Figure 5C), which indicates that these Foxp3^−^CD25^+^ T cells are pathogenic effectors. These results suggest that DCs from DSS-treated Yeti/CD1d KO mice regulate iTreg cell differentiation via proinflammatory cytokines.

### 2.6. CD25^+^CD4^+^ Effector T Cells from DSS-Treated Yeti/CD1d KO Mice Are Pathogenic in DSS-Induced Colitis

**Figure 6:**
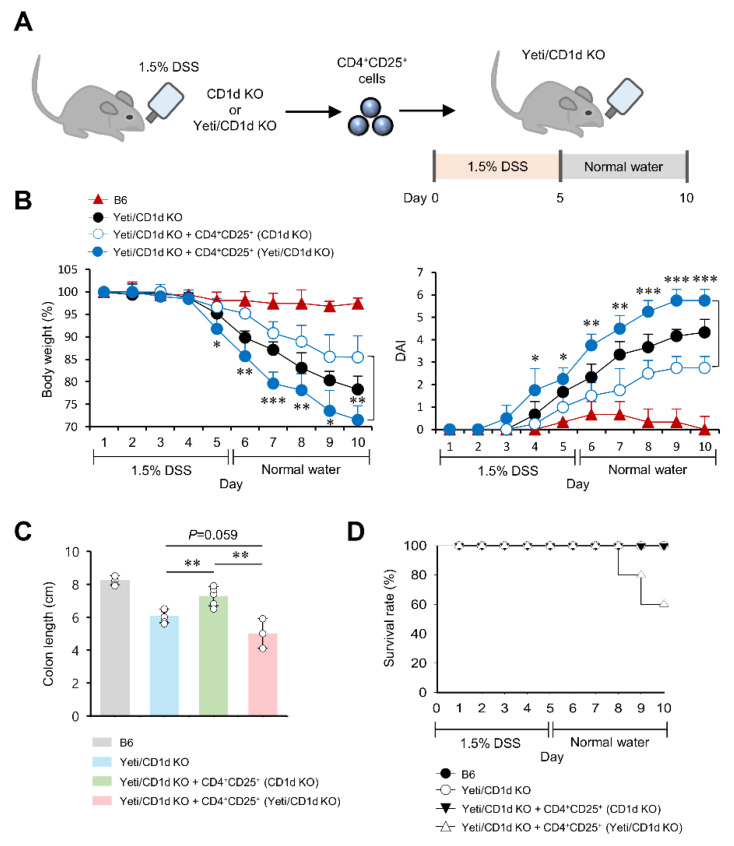
CD25^+^CD4^+^ effector T cells from DSS-treated Yeti/CD1d KO mice are pathogenic in DSS-induced colitis. (**A**–**D**) MLN CD4^+^CD25^+^ T cells (5 × 10^5^) were purified from 1.5% DSS-treated CD1d KO or Yeti/CD1d KO mice and subsequently I.V. transferred to Yeti/CD1d KO mice. Daily body weight changes, disease activity index (DAI) score (**B**), colon length (**C**), and survival rate (**D**) of these mice were evaluated after 1.5% DSS treatment. The mean values ± SD (*n* = 3–5 in (**A**–**C**); *n* = 5 in (**D**); per group in the experiment; Student’s *t*-test; * *p* < 0.05, ** *p* < 0.01, *** *p* < 0.001) are shown. One representative experiment from two experiments is shown.

A previous study demonstrated that downregulation of Foxp3 expression is closely related to the upregulation of pathogenic effector functions and concomitantly decreases the suppressive abilities of CD25^+^CD4^+^ T cells [29]. Therefore, we decided to examine whether MLN CD25^+^CD4^+^ T cells from DSS-treated Yeti/CD1d KO mice (mostly Foxp3^−^ populations) can increase susceptibility to DSS-induced colitis compared to CD25^+^CD4^+^ T cells (primarily Foxp3^+^ populations) from DSS-treated CD1d KO mice. For this purpose, we adoptively transferred MLN CD25^+^CD4^+^ T cells from either DSS-treated CD1d KO mice or DSS-treated Yeti/CD1d KO mice into Yeti/CD1d KO recipient mice. Subsequently, colitis was induced by DSS administration, and disease progression was monitored for ten days (Figure 6A). As expected, adoptive transfer of MLN-derived CD25^+^CD4^+^ T cells from DSS-treated CD1d KO mice into Yeti/CD1d KO recipient mice significantly ameliorated the clinical signs of colitis (i.e., daily body weight loss, disease activity index [DAI], and colon length shortening) compared to the mock-transferred control group (Figure 6B,C). However, the adoptive transfer of MLN-derived CD25^+^CD4^+^ T cells from DSS-treated Yeti/CD1d KO mice into Yeti/CD1d KO recipient mice exacerbated the clinical signs and significantly decreased survival rates compared to the control groups (Figure 6D). These data suggest that the combined effects of both iNKT cell deficiency (CD1d KO) and dysregulated IFNγ production (Yeti) might be attributed to a decrease in protective Foxp3^+^CD25^+^CD4^+^ Treg cells and an increase in pathogenic Foxp3^−^CD25^+^CD4^+^ T cells during DSS-induced colitis.

## 3. Discussion

In this study, we demonstrated that deficiency of iNKT cells in Yeti/CD1d KO mice causes a decrease in Treg cells, with a concomitant increase in pathogenic Foxp3^−^CD25^+^CD4^+^ T cells with Th1 and Th17 phenotypes. Thus, our findings provide strong evidence that MLN-resident iNKT cells possess the capacity to control the differentiation of Foxp3^−^CD25^+^CD4^+^ T cells during DSS-induced colitis. Furthermore, although it has been reported that MLN DCs promote Treg cell differentiation whereas splenic DCs selectively induce Th1 and Th17 differentiation [30], we have shown that MLN DCs from DSS-treated Yeti/CD1d KO mice can induce the differentiation of naive CD4^+^ T cells into effector subtypes with Th1 and Th17 features rather than Treg cells under in vitro Treg polarizing conditions.

A previous study demonstrated that Treg cells can lose Foxp3 expression and suppressive function while gaining T-bet or RORγt expression to become the so-called “ex-Tregs” [31]. It has been proposed that the differentiation of Treg cells into ex-Treg cells requires coordinated alterations in cytokine production within the local microenvironment: (1) loss of IL2/IL10 (cytokines needed for maintaining the Treg population systemically) production, and (2) induction of IL12/IL6 inflammatory cytokine production [31]. In addition, a T cell population referred to as T25 (Foxp3^−^CD25^+^CD4^+^) cells was shown to constitute effector T cells of the Th1 and Th17 lineages, which most likely contain ex-Treg populations [25]. Moreover, it has been reported that the loss of IL10 or TGFβ decreases Foxp3 expression but increases proinflammatory cytokine production by CD25^+^CD4^+^ T cells in the presence of IL2 [29]. These prior studies also raise intriguing questions regarding the mechanisms required to maintain CD25 expression on pathogenic Foxp3^−^CD25^+^CD4^+^ T cells in DSS-treated Yeti/CD1d KO mice. Nish et al. demonstrated that Treg-derived TGFβ diminishes CD25 expression on Foxp3^−^CD25^+^CD4^+^ T cells, whereas proinflammatory cytokines, such as IL6 restore TGFβ-mediated CD25 downregulation by these cells [32]. Since our data showed that MLN DCs exhibit markedly increased expression of IL6 in DSS-treated Yeti/CD1d KO mice, we can speculate that a combined effect of increased proinflammatory cytokine secretion (i.e., IL6) by MLN DCs and decreased TGFβ-producing Treg cells might result in the maintenance of CD25 expression on pathogenic Foxp3^−^CD25^+^CD4^+^ T cells in the MLNs of DSS-treated Yeti/CD1d KO mice. Thus, in future studies, it will be worthwhile to examine whether DC-derived IL6 can maintain Foxp3^−^CD25^+^CD4^+^ T cells that are pathogenic effectors in Yeti mice. Moreover, although MLN DCs in Yeti mice produce high amounts of IL6 irrespective of the presence/absence of iNKT cells, Foxp3 expression by CD25^+^CD4^+^ T cells is maintained in Yeti mice, suggesting that iNKT cells antagonize Foxp3^−^CD25^+^CD4^+^ T cell differentiation, possibly via DC-derived IL6. Thus, in future studies, it will be interesting to compare gene expression profiles of Foxp3^+^CD25^+^ Treg and Foxp3^−^CD25^+^CD4^+^ T cells between Yeti and Yeti/CD1d KO mice.

A phenotypic switch from a regulatory phenotype towards a T-bet-expressing inflammatory phenotype among intestinal Treg cells is closely related to IL2 starvation [33]. In addition, it has been demonstrated that iNKT cell-derived IL2 is critical for regulating Treg cells [34]. Moreover, previous studies have shown that mutual crosstalk between iNKT and Treg cells is critical in regulating allergic diseases, such as asthma [35,36,37]. Furthermore, consistent with previous reports, our studies have shown that CD4^−^CD8^−^ (double negative, DN) iNKT cells protect against allergic skin inflammation in a mouse model of atopic dermatitis, accompanied by the expansion of Treg cells via increased IL2 production [15,16]. However, based on these previous studies, the role of DN iNKT cells in balancing the ratio of Foxp3^+^CD25^+^ Treg cells to Foxp3^−^CD25^+^CD4^+^ T cells during the pathogenesis of colitis remains largely unknown. Thus, in future studies, it will be informative to test the hypothesis that DN iNKT cells are critical for preventing IFNγ-driven colitis pathogenesis by suppressing pathogenic Foxp3^−^CD25^+^CD4^+^ T cell differentiation. Moreover, since the transfer of naive T cells into T cell-deficient mice (i.e., recombinase activating gene-deficient (RAG KO) and TCRβδ KO mice) can induce colitis [38,39], employing these models will be very useful for identifying the precise functions of particular T cell subsets. Thus, further studies will be warranted to investigate the interaction between iNKT cells and Foxp3^−^CD25^+^CD4^+^ T cells using the T cell transfer colitis model.

iNKT cells in the MLN display IL4-producing NKT2 phenotypes and could suppress DSS-induced colitis upon stimulation with orally administered α-galactosylceramide [40]. MLN iNKT cells highly express the Nur77 transcription factor associated with Ag-induced TCR signaling, suggesting that MLN iNKT cells recognize enterogenous bacterial glycolipid antigens in a CD1d/TCR-dependent manner [40,41]. Our result shows that the transfer of Yeti iNKT cells prevents pathogenic MLN Foxp3^−^CD25^+^CD4^+^ T cell differentiation in Jα18 KO recipient mice (they lack iNKT cells but express glycolipid Ag-presenting CD1d), suggesting that Yeti iNKT cells might be activated by glycolipid Ags derived from intestinal commensal bacteria during DSS-induced intestinal inflammation. In addition to MLN iNKT cells, iNKT cells in the colon tissue can recognize CD1d-restricted microbial lipid Ags presented by CD11c-expressing DCs and macrophages, which contributes to maintaining intestinal homeostasis [42]. Furthermore, colonic Treg cells resident in the lamina propria (LP) play an important role in intestinal immune homeostasis [43]. Thus, it will be more informative to further investigate the contribution of iNKT cells to Foxp3 expression by LP CD25^+^CD4^+^ T cells in Yeti mice.

In conclusion, our results demonstrate that the upregulation of pathogenic Foxp3-CD25^+^CD4^+^ T cells induced by proinflammatory DCs is closely linked with increased susceptibility to DSS-induced colitis in iNKT cell-deficient Yeti/CD1d KO mice. Moreover, iNKT cells contribute to protection against IFNγ-mediated colitis by limiting an increase in pathogenic CD25^+^CD4^+^ effector T cells. Thus, our findings identify the crosstalk between iNKT cells and Foxp3^−^CD25^+^CD4^+^ T cells in the MLNs as a novel target for designing IBD immune therapy.

## 4. Materials and Methods

### 4.1. Study Design

This study was designed to determine the combined effect of dysregulated IFNγ expression and iNKT cells on the generation of Treg cells in the MLNs during DSS-induced colitis. For this purpose, Yeti autoinflammatory model mice and iNKT cell-deficient mouse models (CD1d KO or Jα18 KO mice) were employed. During DSS-induced colitis, MLN leukocytes and splenocytes were harvested and further analyzed by flow cytometry. Sejong University Institutional review board approval was obtained before the experiments.

### 4.2. Mice and Reagents

WT mice were purchased from Jung Ang Lab Animal Inc. (Seoul, Republic of Korea). IFNγ/YFP cytokine reporter (Yeti) mice were kindly provided by Dr. R. Locksley (University of California, San Francisco, CA, USA). CD1d KO and Jα18 KO mice were provided by Dr. A. Bendelac (University of Chicago, Chicago, IL, USA) and Dr. M. Taniguchi (RIKEN, Yokohama, Japan), respectively. Yeti mice were further crossed with CD1d KO mice to obtain Yeti/CD1d KO mice. All the mice used in this study were on the B6 genetic background, maintained at Sejong University, and used for experiments at 6–12 weeks of age. Mice were maintained on a 12-h light/12-h dark cycle in a temperature-controlled barrier facility with free access to food and water. Mice were fed a γ-irradiated sterile diet and provided with autoclaved tap water. Age- and sex-matched mice were used for all experiments. The animal experiments were approved by the Institutional Animal Care and Use Committee at Sejong University (SJ-20190301, 3-28-2019).

### 4.3. Induction of Colonic Inflammation

Mice were orally administered 1.5% or 3% of DSS in the drinking water for 5 days. Subsequently, groups of mice were given regular water for five days until they were sacrificed for experiments. To evaluate the clinical symptoms of DSS-induced colitis, the mice were monitored daily for a change in the percentage of body weight (0, none; 1, 1–10%; 2, 11–20%; 3, >20%), stool consistency (0, normal; 1, loose stool; 2, diarrhea), and bleeding (0, normal; 1, hemoccult positive; 2, gross bleeding) during colitis induction for 10 days. The body weight was expressed as a percentage of weight change for each individual mouse and was calculated relative to the starting body weight on day 0. These data were used to calculate the DAI.

### 4.4. Cell Culture and Cell Enrichment by Magnetically Activated Cell Sorting (MACS)

A single-cell suspension of splenocytes and MLN cells was prepared in RPMI complete medium consisting of RPMI 1640 (Gibco BRL, Gaithersburg, MD, USA) medium supplemented with 10% FBS, 10 mM HEPES, 2 mM L-glutamine, 100 units/mL penicillin-streptomycin, and 5μM 2-mercaptoethanol. iNKT cells were enriched using an NK1.1^+^ iNKT cell isolation kit (Miltenyi Biotech, Bergisch Gladbach, Germany) following the manufacturer’s instructions [23]. The NKT cell population was >89% pure among all MACS-purified populations. In addition, CD4^+^CD25^+^ Treg cells and CD11c^+^ DCs were isolated from mice using MACS systems (Miltenyi Biotech, Bergisch Gladbach, Germany), following the manufacturer’s instructions. CD4^+^CD25^+^ Treg cells and CD11c^+^ DC population among all MACS-purified populations were >92% and >95% pure, respectively.

### 4.5. In Vitro Treg Cell Differentiation

Splenocytes were isolated from Jα18 KO mice. Naive CD4^+^CD62L^+^ T cells were separated with a CD4^+^CD62L^+^ T cell isolation kit (Miltenyi Biotech, Bergisch Gladbach, Germany) according to the manufacturer’s instructions. The purified naive CD4^+^CD62L^+^ T cells (1 × 10^5^ cells/well) were incubated in a 96-well plate pre-coated with anti-CD3ε (10 μg/mL) and anti-CD28 (1 μg/mL) mAbs in 10% FBS RPMI media with hTGFβ (10 ng/mL) and mIL2 (100 U/well) for 5 days. MACS-purified CD11c^+^ DCs were added to assess their effect on the generation of Treg cells.

### 4.6. Flow Cytometry

The following mAbs were obtained from BD Biosciences (San Jose, CA, USA): phycoerythrin (PE)-Cy7-, or allophycocyanin (APC)-conjugated anti-CD11c (clone HL3); fluorescein isothiocyanate (FITC)-, PE-Cy7, or APC-conjugated anti-CD4 (clone RM4-5); PE-, PE-Cy7-, or APC-conjugated anti-CD25 (clone PC61); PE-Cy7- or APC-conjugated anti-CD3ε (clone 145-2C11); PE-conjugated anti-IL12p40 (clone C15.6); PE-conjugated anti-TNFα (clone XP6-XT22); PE-conjugated anti-IL6 (clone MP5-20F3); PE-conjugated anti-T-bet (clone 4B10, O4-46); PE-conjugated anti-RORγt (clone Q31-378); and PE-conjugated anti-IgG1 (κ isotype control). In addition, the following mAbs from Thermo Fisher Scientific (Waltham, MA, USA) were used: PE-conjugated anti-IFNγ (clone XMG1.2); PE-conjugated anti-IL17A (clone eBio17B7); and PE-, or PE-Cy7-conjugated anti-Foxp3 (clone NRRF-30). Cells were harvested and washed twice with cold, 0.5% BSA-containing PBS (FACS buffer) for staining surface markers. For blocking Fc receptors, the cells were incubated with anti-CD16/CD32 mAbs (clone 2.4G2) on ice for 10 min and subsequently stained with fluorescently labeled mAbs. Flow cytometric data were acquired using a FACSCalibur flow cytometer (Becton Dickson, San Jose, CA, USA) and analyzed using FlowJo software (version 8.7; Tree Star, Ashland, OR, USA).

### 4.7. Intracellular Cytokine Staining

For intracellular staining, splenocytes were incubated with brefeldin A, an intracellular protein transport inhibitor (10 μg/mL), in RPMI complete medium for 2 h at 37 °C. The cells were stained for cell surface markers, fixed with 1% paraformaldehyde, washed once with cold FACS buffer, and permeabilized with 0.5% saponin. The permeabilized cells were then stained for an additional 30 min at room temperature with the indicated mAbs (PE-conjugated anti-IFNγ, anti-IL12, anti-IL6, anti-TNFα, anti-IL17, or PE-conjugated isotype control rat IgG mAbs). Fixation and permeabilization were performed using a Foxp3 staining kit (eBioscience, San Diego, CA, USA) with the indicated mAbs (PE-conjugated anti-Foxp3, anti-T-bet, anti-RORγt, or isotype control rat IgG mAbs) [44]. More than 5000 cells per sample were acquired using a FACSCalibur, and the data were analyzed using the FlowJo software package (Tree Star, Ashland, OR, USA).

### 4.8. Statistical Analysis

Statistical significance was determined using Excel software (Microsoft, Redmond, WA, USA). Student’s *t*-test was performed to compare two groups. In the Student’s *t*-test, * *p* < 0.05, ** *p* < 0.01, and *** *p* < 0.001 were considered significant. Two-way ANOVA analysis was carried out using the VassarStats (http://vassarstats.net/anova2u.html) (accessed on 19 January 2022). In the two-way ANOVA, ^#^ *p* < 0.05, ^##^ *p* < 0.01, and ^###^ *p* < 0.001 were considered significant.

## Data Availability

The data will be available from the corresponding author upon reasonable request.

## Supplementary Materials

The following supporting information can be downloaded at: https://www.mdpi.com/article/10.3390/ijms232315316/s1.
