# Supplementary material for: Opposing Roles of DCs and iNKT Cells in the Induction of Foxp3 Expression by MLN CD25+CD4+ T Cells during IFNγ-Driven Colitis
Source: Int J Mol Sci. 2022 Dec 5;23(23):15316. doi: 10.3390/ijms232315316 (PMC9738888; doi:10.3390/ijms232315316)
Supplement: Supplementary file 1 [file ijms-23-15316-s001.zip › ijms-1995522-supplementary.pdf]

Supplementary Materials to:

Opposing roles of DCs and iNKT cells in the induction of Foxp3 expression by MLN $CD25^+CD4^+$ T cells during IFNγ-driven colitis

This document includes:

-Supplementary figures 1-3

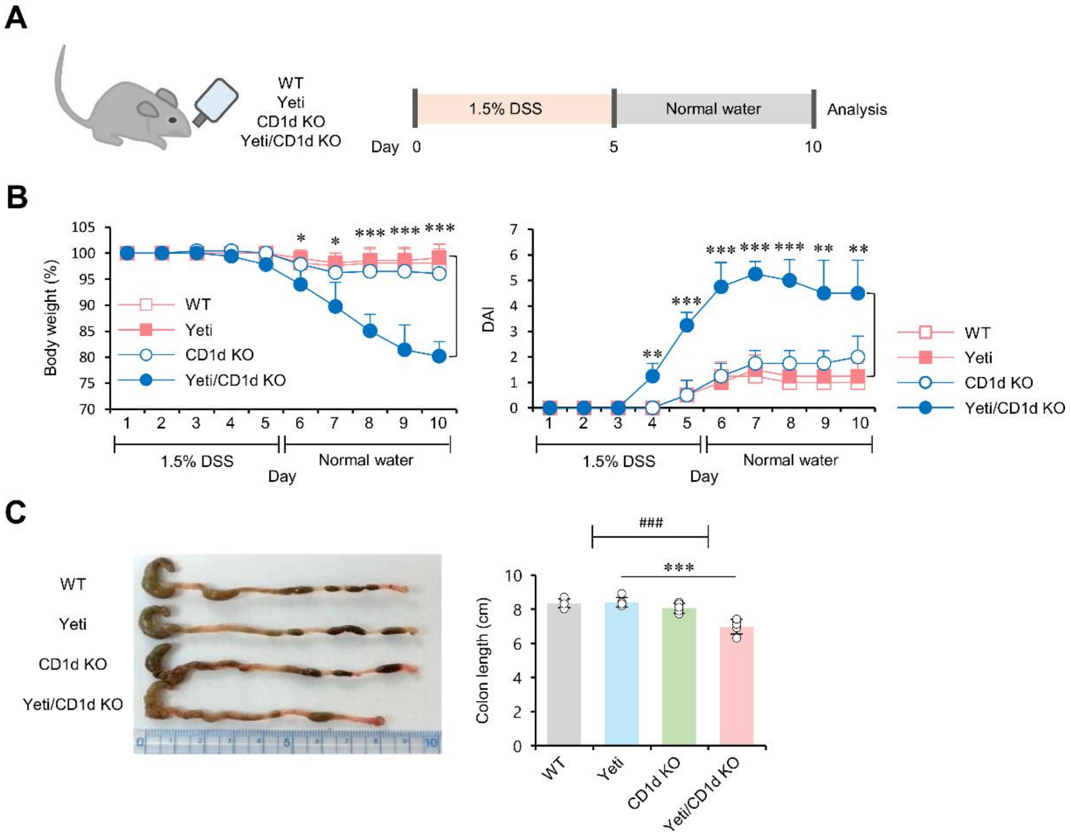

Figure S1. iNKT cells protect Yeti mice from DSS-induced colitis.

(A) Experimental outline. WT, Yeti, CD1d KO, and Yeti/CD1d KO mice were treated with 1.5% DSS as described in materials and methods. Daily body weight changes, DAI score (B), and colon length (C) of WT, Yeti, CD1d KO, and Yeti/CD1d KO mice were evaluated after 1.5% DSS treatment. The mean values ± SD (n = 5; per group in the experiment; Student's t-test; * $P < 0.05$, ** $P < 0.01$, *** $P < 0.001$) are shown. Two-way ANOVA (Yeti × iNKT) showed an interaction between these two factors ($^{###}$ $P < 0.001$). One representative experiment of two experiments is shown.

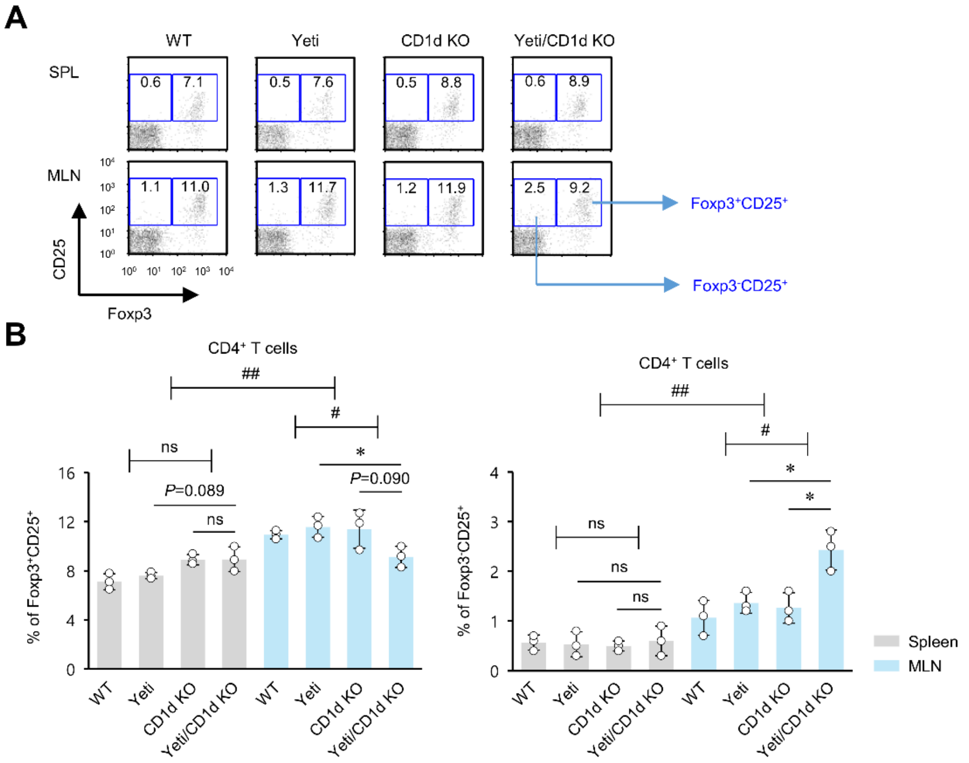

Figure S2. Foxp3 expression by CD25^{+}CD4^{+} T cells in the spleen and MLNs of Yeti/CD1d KO mice under steady-state conditions.

The spleen and mesenteric lymph nodes (MLN) were collected from WT, Yeti, CD1d KO, and Yeti/CD1d KO mice. The percentages of Foxp3^{+}CD25^{+} and Foxp3^{-}CD25^{+} subpopulations gated on CD4^{+} T cells from the spleen and MLNs of each group were measured by flow cytometry. Representative data (A) and their summary (B) are shown. The mean values ± SD (n = 3; per group in the experiment; Student's t-test; * $P < 0.05$) are shown. Two-way ANOVA (Yeti × iNKT) showed an interaction between these two factors ($^{\#}$ $P < 0.05$). Two-way ANOVA (genotype × tissue) showed an interaction between these two factors ($^{\#\#}$ $P < 0.01$). ns, not significant.

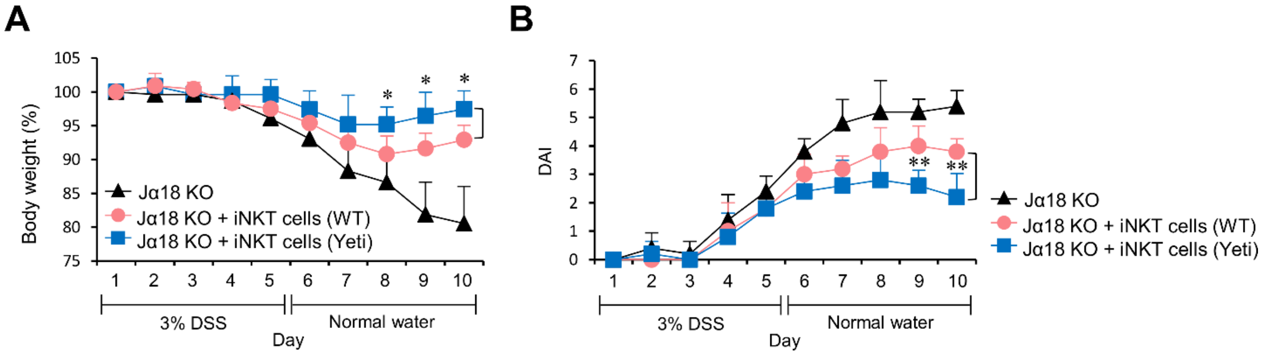

Figure S3. The suppressive effects of MLN iNKT cells on IFNγ-mediated intestinal inflammation.

iNKT cell-deficient Jα18 KO mice were adoptively transferred with either WT or Yeti MLN iNKT cells (2×10^5) and subsequently treated with 3% DSS as described in Materials and Methods. (A) Daily body weight changes and (B) DAI scores of these mice were evaluated. The mean values ± SD ($n = 3$; per group in the experiment; Student's t-test; * $P < 0.05$, ** $P < 0.01$) are shown. One representative experiment of two experiments is shown.
